# Nephroprotective effects of visnagin through modulation of macrophage polarization, oxidative stress, inflammation and apoptosis in renal I/R injury

**DOI:** 10.1515/med-2026-1399

**Published:** 2026-03-09

**Authors:** Suleyman Sagir, Ugur Seker, Merve Pekince-Ozoner, Meral Yuksel, Gul Sahika Gokdemir, Seval Kaya, Mehmet Demir

**Affiliations:** Department of Urology, Faculty of Medicine, Mardin Artuklu University, Mardin, Türkiye; Department of Histology and Embryology, Faculty of Medicine, Mardin Artuklu University, Madin, Türkiye; Department of Veterinary Histology and Embryology, Faculty of Veterinary Medinice, Siirt University, Siirt, Türkiye; Department of Medical Laboratory, Vocational School of Health-Related Professions, Marmara University, İstanbul, Türkiye; Department of Physiology, Faculty of Medicine, Mardin Artuklu University, Mardin, Türkiye; Department of Histology and Embryology, Faculty of Medicine, İstanbul Aydın University, İstanbul, Türkiye; Department of Urology, Faculty of Medicine, Harran University, Şanlıurfa, Türkiye

**Keywords:** visnagin, ischemia-reperfusion injury, macrophage polarization, oxidative stress, inflammation, apoptosis

## Abstract

**Objectives:**

The study aimed to investigate the nephroprotective effects of visnagin on renal ischemia-reperfusion (I/R) injury and the role of M1/M2 macrophage polarization in this process.

**Methods:**

Forty-two adult rats were divided into six groups: Control, Visnagin30 mg/kg, Visnagin60 mg/kg, I/R, I/R + Visnagin30 mg/kg, I/R + Visnagin60 mg/kg (n=7). Bilateral renal ischemia was induced by clamping for 25 min, followed by 2 h of reperfusion. Visnagin or vehicle was administered to the animals intraperitoneally 2 h before reperfusion. At the end of the study, kidney samples were collected for analysis of oxidative stress, inflammatory cytokines, apoptotic protein expression, and M1/M2 macrophage polarization.

**Results:**

I/R injury increased malondialdehyde (MDA), chemiluminescence (CL), IL-1β, and IL-6 levels while decreasing glutathione (GSH) in renal tissue, indicating enhanced oxidative stress (p<0.001) and inflammation (p<0.05). Histopathological examination showed glomerular atrophy, tubular degeneration, and intertubular hemorrhage. Visnagin treatment at 60 mg/kg significantly reduced MDA, CL, and IL-1β levels, and increased GSH (p<0.05). Immunohistochemically, visnagin decreased Bax (p<0.001), caspase-3 (p<0.01), and TNF-α (p<0.01) expressions elevated by I/R injury. Furthermore, visnagin reversed I/R induced M1/M2 macrophage polarization (CD86↑, CD163↓), decreasing CD86 (p<0.05) and increasing CD163 immunodensity (p<0.05).

**Conclusions:**

Visnagin treatment (60 mg/kg) exerts promising nephroprotective effects in renal I/R injury by reducing oxidative stress, inflammation, apoptosis, and modulating M1/M2 macrophage polarization.

## Introduction

The temporary interruption of renal blood flow is a condition encountered kidney transplantation, partial nephrectomy, aortic bypass surgery, accidental or iatrogenic trauma, sepsis, hydronephrosis, and anatrophic nephrolithotomy, where the blood flow to the kidneys is temporarily obstructed [1]. Reperfusion is essential for the revival of ischemic renal tissue; however, following reperfusion, the renal tissue becomes further complicated by additional damage due to the formation of oxygen-derived free radicals [2]. The underlying mechanisms of ischemia-reperfusion (I/R) injury in the kidneys are likely multifactorial and interdependent, involving hypoxia, vascular endothelial damage, inflammatory responses, free radical-induced injury, apoptosis, and endothelial cell damage [3]. Numerous studies have highlighted the protective and free radical-neutralizing effects of antioxidant substances, establishing their significance in renal I/R injury [4], [5], [6]. Most of these studies have demonstrated promising results of the protective effects of antioxidants on the kidney during I/R injury [7].

Macrophages function in two basic phenotypes during kidney injury and recovery: M1 (pro-inflammatory) and M2 (anti-inflammatory). M1 macrophages are activated by stimuli such as lipopolysaccharide (LPS) and interferon-gamma (IFN-γ) and secrete mediators such as TNF-α, IL-1β, IL-6, and NO, thereby increasing inflammation and tissue damage. In contrast, M2 macrophages are activated by stimuli such as IL-4 and IL-13 and release anti-inflammatory cytokines such as IL-10 and TGF-β, supporting resolution of inflammation and tissue repair. CD86 is a surface marker for M1 macrophages, while CD163 is specific to M2 macrophages [8], 9]. In recent years, it has been shown that macrophages can switch between M1 and M2 phenotypes depending on environmental signals, and this process has been defined as “macrophage polarization” [10], 11]. In an environment dominated by M1 macrophages, CD86 expression is high, whereas in an environment dominated by M2 macrophages, this expression shifts toward CD163 [12]. The balance between these two macrophage types is crucial for regulating inflammation and optimizing tissue healing, as macrophages are major sources of reactive oxygen and nitrogen species release [13]. Therefore, Visnagin’s potential to regulate macrophage polarization and oxidative stress pathways may explain the kidney-protective effect observed in the I/R injury model.

*Ammi visnaga* is a plant widely used in traditional medicine, particularly in the Middle East and North Africa, as a diuretic, antispasmodic, and kidney stone treatment. It is known to alleviate symptoms of renal colic, particularly in Egypt, when used as a fruit tea. Modern *in vitro* and animal studies also support the validity of this traditional use; *A. visnaga* seed extract has shown a protective effect against nephrotoxicity by reducing oxidative stress and inflammation in CCl_4_-induced renal damage [14]. Visnagin is a compound derived from the extract of the *A. visnaga* plant and is known as 4,9-dimethoxy or 4-methoxy-7-methyl-furo[3,2-g]chromen-5-one [15]. Moreover, Visnagin has been shown to reduce oxidative stress, protect against oxalate-induced damage in renal epithelial cells, and exhibit protective effects against isoproterenol-induced acute myocardial injury [16], 17]. It has also been demonstrated that N-isopropylacrylamide-methacrylic acid nanoparticles containing Visnagin prevent I/R associated cardiac damage and dysfunction in rats by inhibiting apoptosis and induction of autophagy [18]. The inhibition of apoptosis contributes to reducing cell death during the I/R process, thereby minimizing damage in cardiac tissue and preserving its functions. These findings suggest that visnagin, administered in nanoparticle form, could be effective in the treatment of I/R injury [18]. Our study highlights the impact of visnagin treatment on M1/M2 macrophage polarization in renal ischemia-reperfusion injury. While current literature emphasizes the protective effects of various agents that reduce oxidative stress and inflammation in renal I/R injury, studies focusing on macrophage polarization are limited.

For that reason, in this study, we aimed to examine the protective potential of visnagin on renal I/R induced oxidative stress, inflammation, apoptosis and understating the pivotal role of M1/M2 macrophage polarization during this process.

## Methods

### Study design and experimental protocol

The obtained 42 male Wistar albino rats (200–250 g) were housed in a room with 12 h light and dark cycles, with temperature (22 ± 2 °C), relative humidity (65–70 %) and divided into six groups as follows: Control, Visnagin30, Visnagin60, I/R, I/R + Visnagin30, I/R + Visnagin60 (each group n=7). The animals in I/R, I/R + Visnagin30, and I/R + Visnagin60 groups were exposed to bilateral renal ischemia model [19]. Briefly, renal ischemia in I/R, I/R + Visnagin30, and I/R + Visnagin60 groups was induced by clamping the renal pedicle in animals under general anesthesia [20], 21]. The blood flow to the kidneys were ceased in these groups for 25 min. Following the ischemia, the animals exposed to 2 h of reperfusion. The reperfusion duration is determined with considering methodological protocols of a previous study [22]. The animals in the control, Visnagin30 and Visnagin60 groups received a laparotomy without an occlusion. The animals in Visnagin30, Visnagin60, I/R + Visnagin30 and I/R + Visnagin60 groups received a single dose of 30 or 60 mg/kg *intraperitoneally* Visnagin according to the group’s name 2 h before the reperfusion because of the pharmacokinetic studies demonstrated that the plasma level of the Visnagin reaches to the highest plasma levels at the 2 nd h of exposure [23]. Powder form of Visnagin is dissolved in DMSO and diluted 10 times with distilled water before administration. The animals in Visnagin30, Visnagin60 groups received equal volume of DMSO diluted in distilled water as vehicle. At the end of the experimental procedure, all of the animals were sacrificed with cardiac exsanguination under general anesthesia with administration of xylazine (10 mg/kg) and ketamine (50 mg/kg). One of the kidneys from each animal were fixed in 10 % for histopathological examinations and the contralateral samples were frozen at −80 °C for biochemical examinations.

### Determination of the dose of visnagin

Visnagin (Cayman Chemical, MI, USA); Catalog no: 34140 doses were selected based on a previous study evaluating the dose-response relationship in testicular ischemia-reperfusion models. Sagır et al. reported that visnagin administered at doses of 30 mg/kg and 60 mg/kg significantly reduced testicular I/R injury and provided statistically significant improvements in oxidative stress, inflammation, and apoptotic protein expression [15]. Therefore, the 30 mg/kg and 60 mg/kg doses were chosen to facilitate comparisons between different doses in renal I/R model.

### Measurement of serum BUN and creatinine levels

Blood samples collected from each animal were centrifuged (Megafuge STPlus, Thermo Scientific, Waltham, MA) at 1,500 g for 10 min. Serum supernatants were collected in sterile tubes, and BUN and creatinine levels were analyzed on a biochemical autoanalyzer (Architect c8000; Abbott, Wiesbaden, Germany). Both of the BUN and creatinine level results are expressed as mg/dL.

### Determination of renal oxidative stress

Tissue oxidative stress was evaluated with considering malondialdehyde (MDA), glutathione (GSH) levels, luminol and lucigenin enhanced chemiluminescence (CL) assay [24]. Tissue MDA content were measured for products of lipid peroxidation via the reaction of thiobarbituric acid- reactive substances, and the endogenous antioxidant GSH levels were determined using Ellman’s reagent. Luminol enhanced CL was performed to measure the release of hypochlorous acid radicals, hydroxyl radicals, and hydrogen peroxide reactive species. On the other hand, the amount of superoxide radical generation was measured using the lucigenin enhanced CL method. The results of the CL measuremend were expressed as rlu/mg, while MDA and GSH levels were reported as nmol/g and μmol/g, respectively.

### Determination of renal IL-1β and IL-6 levels

Tissue IL-1β and IL-6 levels were measured using the enzyme-linked immunosorbent assay (ELISA) tests. For that purpose, ready-to-use sandwich ELISA kits were employed to measure tissue IL-1β (Sunred Biotechnology, Baoshan, Shanghai, CH, Rat IL-1β ELISA Kit, Catalog no: 201-11-0120) and IL-6 (Sunred Biotechnology, Baoshan, Shanghai, CH, Rat IL-6 ELISA Kit, Catalog no: 201-11-0136) levels. Before the ELISA Assay, total protein levels in the homogenized tissue samples were measured using the SMART™ BCA Protein Assay Kit (iNtRON Biotechnology DR, 137 Gyeonggi, KR, Catalog no: 21071) in which the homogenates were prepared according to the manufacturer’s instructions. The protocols for the ELISA kits were carried out according to the manufacturer’s instructions, and the absorbance of the prepared samples was measured at a wavelength of 450 nm using a microplate reader (Rel Assay Diagnostics, Gaziantep, TR). The obtained optical density (OD) values were evaluated using a standard curve generated from known standards, and tissue IL-1β and IL-6 levels were determined. Both IL-1β and IL-6 levels were expressed as picograms per milligram of protein (pg/mg protein).

### Histopathologic preparation and analyses

Tissue processing and steps were carried out as previously described [25]. Briefly, tissues fixed in 10 % formalin were washed in tap water, dehydrated in increasing concentrations of alcohol, cleared in xylene, and embedded in paraffin blocks. Sections 5 µm thick were cut from the samples using a rotary microtome and stored for histopathological and immunohistochemical analysis. For routine histopathological examination, the sections were deparaffinized in xylene, rehydrated through decreasing alcohol series, and washed in distilled water before being stained with hematoxylin and eosin. All histological sections were examined, micrographs were taken and all the evaluations were performed under a light microscope.

### Immunohistochemistry staining and immunodensity analyses

Immunohistochemistry was performed on sections obtained from tissue embedded in paraffin blocks to evaluate the immunoexpression of pro-apoptotic Bax (Santa Cruz Biotechnology, Dallas, TX, USA; Catalog no: sc-7480), Caspase 3 (Santa Cruz Biotechnology, Dallas, TX, USA; Catalog no: sc-56053), pro-inflammatory TNF-α (Santa Cruz Biotechnology, Dallas, TX, USA; Catalog no: sc-52746), and M1/M2 macrophage polarization markers CD86 (Santa Cruz Biotechnology, Dallas, TX, USA; Catalog no: sc-28347) and CD163 (Santa Cruz Biotechnology, Dallas, TX, USA; Catalog no: sc-58965) [26]. The samples were deparaffinized in xylene, rehydrated through a graded series of alcohols, and washed in PBS. Antigen retrieval was performed by heating the samples in citrate buffer (pH 6.0). Following antigen retrieval, the sections were incubated in 3 % H_2_O_2_ for 15 min to inhibit endogenous peroxidase activity. After washing in PBS, the samples were incubated with a blocking solution to prevent nonspecific antibody binding. Subsequently, the sections were incubated overnight with primary antibodies against Bax, Caspase 3, TNF-α, CD86, and CD163. For subsequent steps, a ready-to-use immunohistochemistry kit (Thermo Scientific, Waltham, MA, USA; Catalog no: TP-125-HL) and a DAB chromogen kit (Thermo Scientific, Waltham, MA, USA; Catalog no: TA-125-HD) were used for secondary antibody, enzyme, and chromogenic reactions. The sections were counterstained with hematoxylin, mounted with Entellan, and the expression areas of the relevant proteins were examined under a light microscope. Additionally, immunodensity analysis was performed on the prepared immunohistochemistry slides to quantify the immunodensity of Bax, Caspase 3, TNF-α, CD86, and CD163 proteins. For this purpose, 28 randomly selected fields from each group for each immunostaining were analyzed. The DAB-positive areas were measured and compared to the total section area using ImageJ software (National Institutes of Health, Bethesda, MD, USA; Version: 1.54j). The obtained values were used for immunodensity measurements [27]. Briefly total DAB positive areas are and the total tissue sections were measured with Image J software and the obtained results were converted to percentage manually.

### Statistical analysis

All obtained data in this study were examined statistically. For that purpose, a normality test of Kolmogorov-Smirnov was performed to determine if the datasets are distributed normally or not. The statistical analyses were performed with Statistical Package for the Social Sciences – Software Version 24.0 (IBM, NY, USA). Due to determination of normally distribution, parametric One-Way ANOVA was used for statistical analysis and multiple comparisons between the groups is performed with post-hoc Tukey test. The results are expressed as mean±standard deviation (SD) and results are considered significant when the p value was lower than 0.05.

### Ethical approval

The animals used in this study are received from Harran University Experimental Animal Research Unit with the approval of the Experimental Animal Ethics Committee of Harran University (Approval date and number: 10.05.2023, 217188). All experimental protocols were conducted in accordance with ARRIVE (Animal Research: Reporting of *in Vivo* Experiments) guidelines. Animal welfare was prioritized throughout the study; humane outcome criteria were applied to minimize pain, stress, and suffering, and veterinary supervision was maintained throughout the experiment.

## Results

### Serum BUN and creatinine results

**Figure 1:**
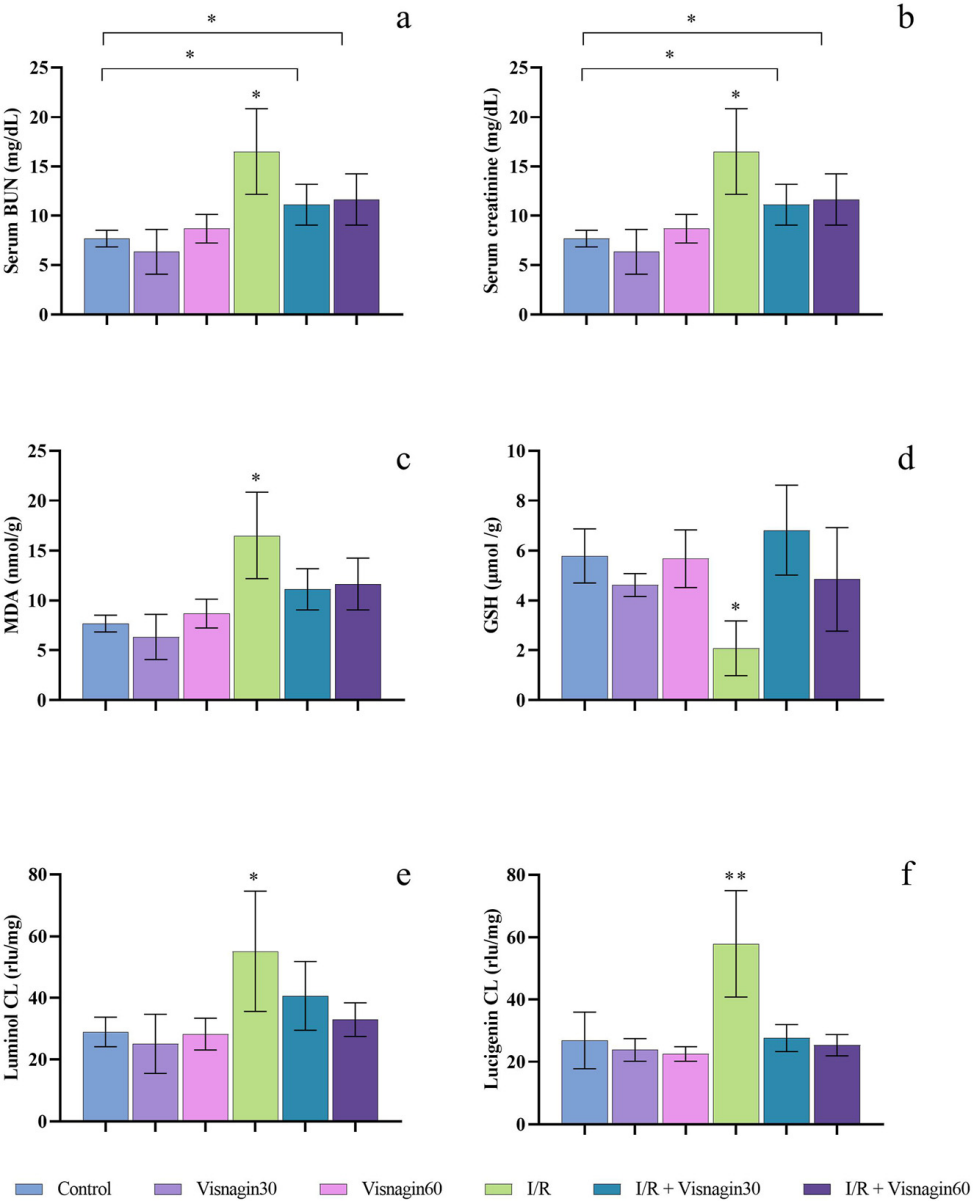
Graphical demonstration of the serum BUN (a) and creatinine (b), and tissue MDA (c), GSH (d), luminol CL (e), lucigenin CL (f) results. The existence of superscripts on bar graphs indicate statistically significance. *p<0.05, **p<0.001 compared to control group. Existing asterisk between the groups control and I/R + Visnagin30 or I/R + Visnagin60 groups indicates statistically significance (p<0.05).

BUN level in Control group was 28.29 ± 0.76 mg/dL. The results in Visnagin30 and Visnagin60 groups were 28.14 ± 0.69 mg/dL and 28.00 ± 0.82 mg/dL respectively. Statistical results indicated no significant difference (p>0.05) among these groups. However, the BUN level in I/R group is elevated to 29.86 ± 0.90 mg/dL and results of this group was significantly different (p<0.05) compared to Control, Visnagin30 and Visnagin60 groups. The BUN levels in I/R + Visnagin30 and I/R + Visnagin60 groups were 29.86 ± 0.69 mg/dL and 29.57 ± 0.79 mg/dL. Results of these groups were similar (p>0.05) to each other and I/R group. In addition, the serum BUN level of I/R, I/R + Visnagin30 and I/R + Visnagin60 groups were significantly different from Control, Visnagin30 and Visnagin60 groups although the statistical differences were close to similarity (Figure 1a).

When the serum creatinine levels (mg/dL) were considered, Control group’s result was 0.89 ± 0.01 mg/dL. The results in Visnagin30 and Visnagin60 groups were measured as 0.88 ± 0.01 mg/dL and 0.88 ± 0.02 mg/dL. The creatinine level in experiment group of I/R was 0.92 ± 0.01 mg/dL and results of this group was significantly different compared to Control, Visnagin30 and Visnagin60 groups. Moreover, the serum levels of I/R + Visnagin30 and I/R + Visnagin60 groups were detected as 0.91 ± 0.02 mg/dL and 0.91 ± 0.02 mg/dL respectively. Results of these two treatment groups were similar (p>0.05) to the I/R group and each other. The statistical analyses indicated that the serum creatinine level of Control, Visnagin30 and Visnagin60 were significantly different (p<0.05) than I/R, I/R + Visnagin30 and I/R + Visnagin60 groups (Figure 1b).

### Renal tissue oxidative stress analyses results

The levels of MDA (Figure 1c), measured as an indicator of lipid peroxidation, were significantly higher in the I/R group compared to the control group (p<0.001). Both doses of Visnagin treatment significantly reduced MDA formation in renal tissues (p<0.01 for I/R + Visnagin30 and p<0.05 for I/R + Visnagin60). However, the renal GSH content (Figure 1d) after I/R injury was significantly decreased compared to the vehicle-treated control group (p<0.001). Visnagin treatments increased GSH levels in renal tissues after I/R at both doses (p<0.001 for I/R + Visnagin30 and p<0.05 for I/R + Visnagin60). Luminol and lucigenin-enhanced CL measurements (Figure 1e and f) were significantly elevated in the renal tissues of the I/R-injured group compared to the control group (p<0.01 and p<0.001, respectively). Treatment with Visnagin significantly reduced superoxide radical generation, as measured by the lucigenin probe, at both doses (p<0.001 for I/R + Visnagin30 and I/R + Visnagin60). But luminol-enhanced CL measurements was decreased significantly (p<0.05) only in I/R + Visnagin60 group. The exact results of the tissue MDA, GSH, luminol and lucigenin-enhanced CL Assay are presented in Table 1.

**Table 1:** Statistical analysis results of MDA, GSH, luminol and lucigenin-enhanced CL, IL-1β and IL-6 levels in renal tissues of the groups.

	Control	Visnagin30	Visnagin60	I/R	I/R + Visnagin30	I/R + Visnagin60
MDA, nmol/g	7.69 ± 0.85	6.35 ± 2.27	8.69 ± 1.45	16.52 ± 4.32^b^	11.13 ± 2.07^++^	11.66 ± 2.61^+^
GSH, μmol/g	5.79 ± 1.09	4.62 ± 0.46	5.68 ± 1.16	2.08 ± 1.10^b^	6.82 ± 1.80^+++^	4.85 ± 2.08^+^
Luminol CL, rlu/mg	28.97 ± 4.80	25.10 ± 9.57	28.25 ± 5.15	55.07 ± 19.47^a^	40.65 ± 11.16	32.97 ± 5.47^+^
Lucigenin CL, rlu/mg	26.88 ± 9.10	23.80 ± 3.66	22.52 ± 2.34	57.85 ± 17.03^b^	27.62 ± 4.35^+++^	25.35 ± 3.43^+++^
IL-1β, pg/mg	795.06 ± 228.60	785.4 ± 143.41	628.33 ± 300.65	1760.85 ± 641.09^a^	1255.95 ± 756.19	849.00 ± 407.56^++^
IL-6, pg/mg	32.13 ± 11.62	34.21 ± 11.49	30.23 ± 9.14	115.99 ± 77.98^a^	70.64 ± 35.50	57.14 ± 20.21

Data are shown as Mean±SD ^a^p<0.01, ^b^p<0.001 vs. respective control group. ^+^p<0.05, ^++^p<0.01, ^+++^p<0.001 vs. respective I/R group.

### Tissue IL-1β and IL-6 level results

Renal IL-1β and IL-6 levels are significantly higher in I/R injured group (p<0.01 for both) with respect to the control group. Visnagin treatment with 60 mg/kg reduced the IL-1β levels in I/R + Visnagin60 group significantly (p<0.01). Treatment with 30 mg/kg Visnagin reduced both IL-1β and IL-6 levels in I/R + Visnagin30 group but the results were not significant (p>0.05). There was no significance between the control group and Visnagin30 and Visnagin60 groups in MDA, GSH, luminol/lucigenin-enhanced CL and IL-1β and IL-6 levels (p>0.05). The exact results of the tissue ELISA analyses of IL-1β and IL-6 are presented in Table 1.

### Histopathological results

**Figure 2:**
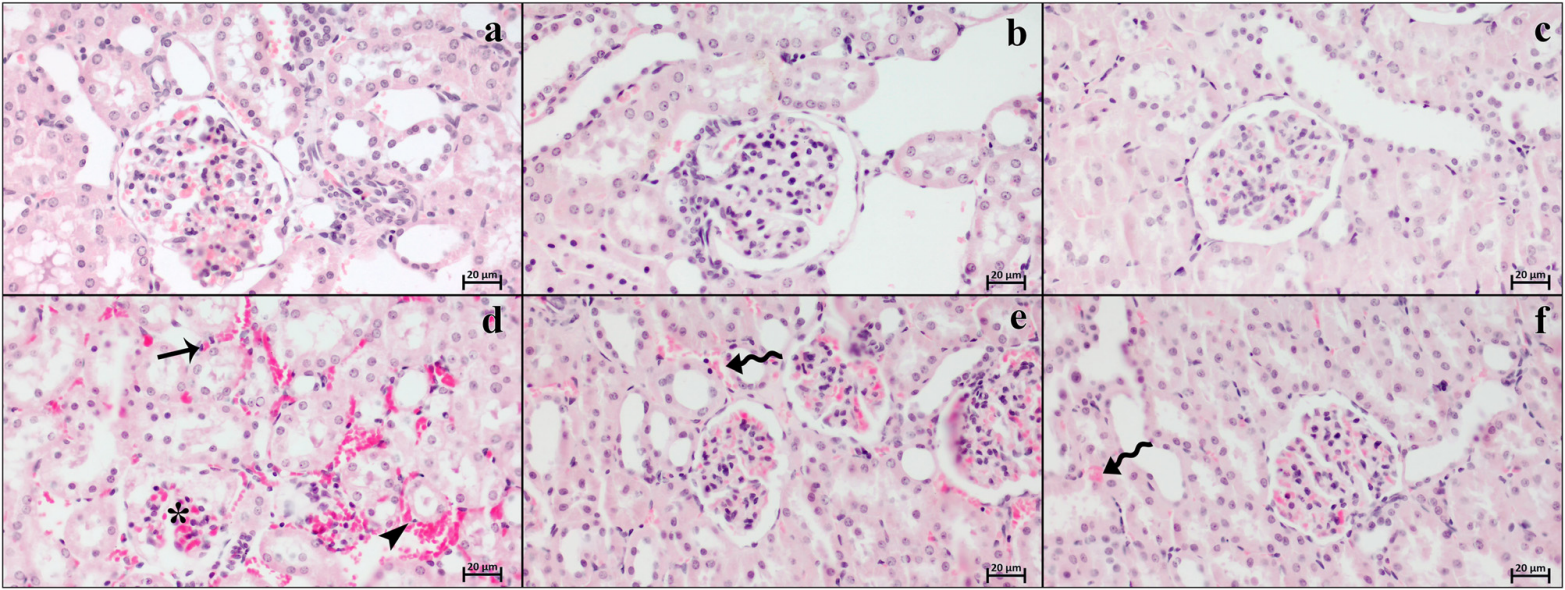
Representative histopathology micrographs of control (a), Visnagin30 (b), Visnagin60 (c), I/R (d), I/R + Visnagin30 (e), and I/R + Visnagin60 (f) groups. The normal glomerular and tubular structure in control, Visnagin30 and Visnagin60 groups. In I/R group, intertubular hemorrhage (arrow), atrophic glomerulus with pyknotic cell nuclei and accumulation of peripheral blood within the glomerular capillaries (asterisk), degenerated tubular cells with pyknotic nuclei and even desquamation of tubular cells into the lumens (arrowhead). In addition, the degenerations alleviated in I/R + Visnagin30 group except the widespread but alleviated hemorrhage (curved arrow), but in I/R + Visnagin60 group the mentioned hemorrhage is almost disappeared (curved arrow) and renal structure was more similar to the control group. Staining: Hematoxylin and eosin. Bar: 20 µm.

Light microscopic examinations revealed that the normal renal tissue organization was observed in the control (Figure 2a), Visnagin30 (Figure 2b), and Visnagin60 (Figure 2c) groups. The kidney was surrounded by perirenal adipose tissue, with a capsule immediately adjacent to the renal cortex. The cellular characteristics of the renal tubules and corpuscles were easily distinguishable, and the renal vascular structures maintained normal arterial portal circulation between the tubules and corpuscles (Figure 2a). The renal morphology in the Visnagin30 and Visnagin60 groups was also found to be similar to that of the control group (Figure 2b and c, respectively). In contrast, the I/R group exhibited glomerular atrophy in the renal corpuscle structure, pyknosis in parietal cells, and significant hemorrhage and degeneration in the intertubular area. The proximal and distal tubules of the I/R group showed localized intense edema accumulation, atrophy and nuclear pyknosis in tubular cells, and edema within the tubular lumen (Figure 2d). In the I/R + Visnagin30 group, the pathological changes were partially mitigated, although intertubular hemorrhage was still occasionally observed. However, the morphology of the corpuscles, glomeruli, and tubular cells was more similar to that of the control group (Figure 2e). In the I/R + Visnagin60 group, except for some hemorrhagic areas, the overall morphology was found to be more similar to that of the control group (Figure 2f).

### Immunohistochemistry and immunodensity results

**Figure 3:**
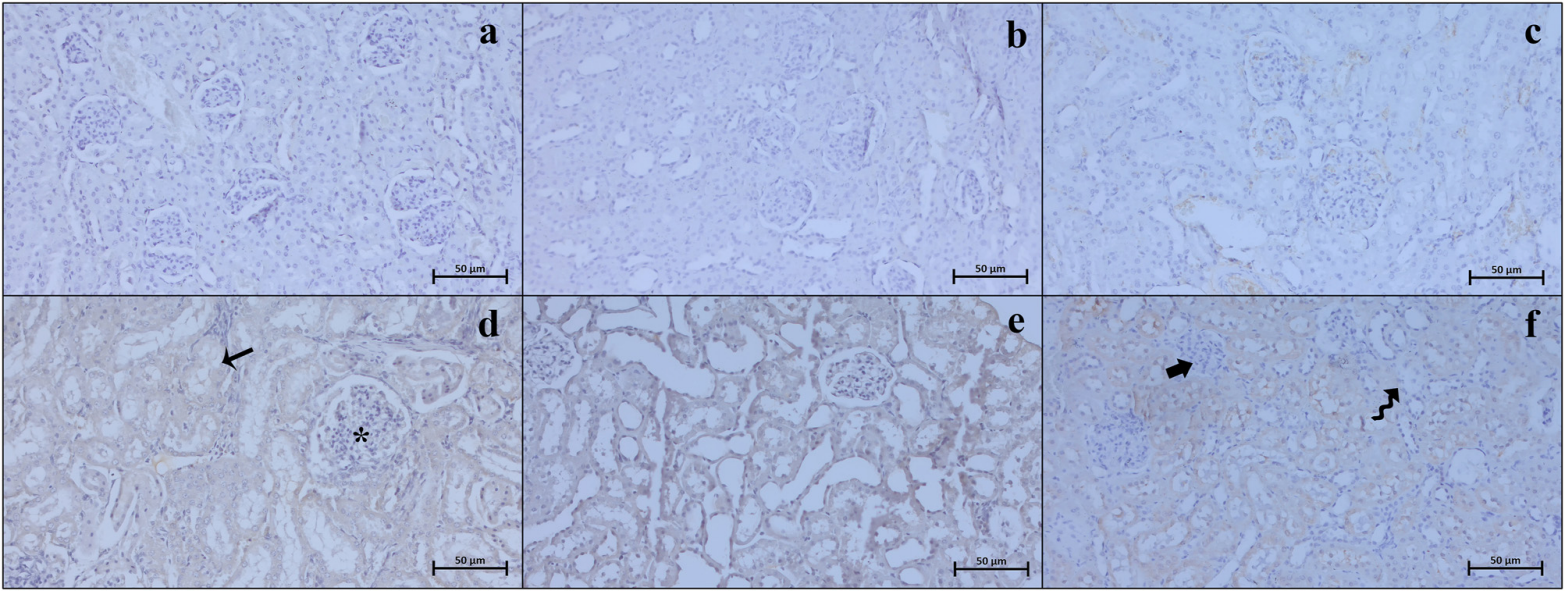
Representative Bax immunohistochemistry micrographs of control (a), Visnagin30 (b), Visnagin60 (c), I/R (d), I/R + Visnagin30 (e), and I/R + Visnagin60 (f) groups. Increased Bax immunodensity in renal tubule (arrow) and glomerular (asterisk) structure reduced in tubules (curved arrow) and glomerulus (thick arrow) in visnagin treated animals. Staining: Bax IHC. Counterstain: Hematoxylin. Bar: 50 µm.

**Figure 4:**
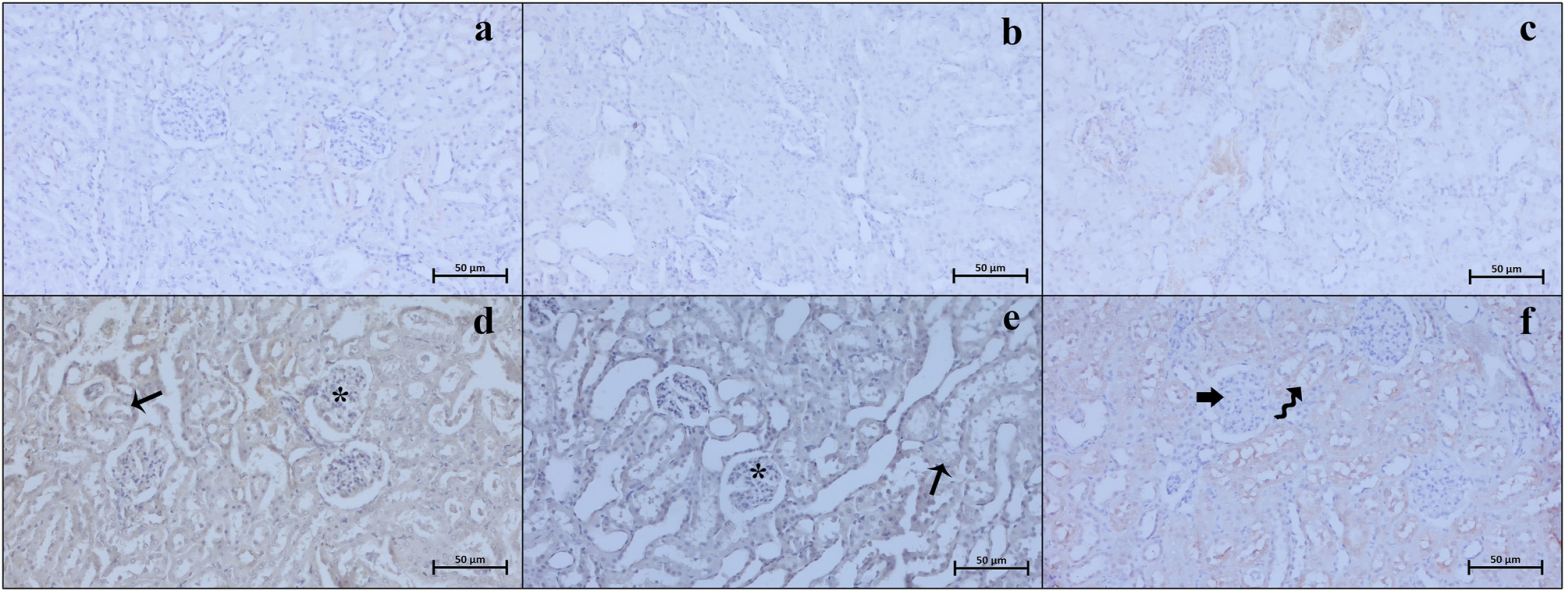
Representative Caspase 3 immunohistochemistry micrographs of control (a), Visnagin30 (b), Visnagin60 (c), I/R (d), I/R + Visnagin30 (e), and I/R + Visnagin60 (f) groups. Caspase 3 immunoexpression is elevated in tubular (arrow) and renal glomerular cells (asterisk) of I/R group. The immunodensity changed in tubule (curved arrow) and glomerulus (thick arrow) in Visnagin treated animals. Staining: Caspase 3 IHC. Counterstain: Hematoxylin. Bar: 50 µm.

**Figure 5:**
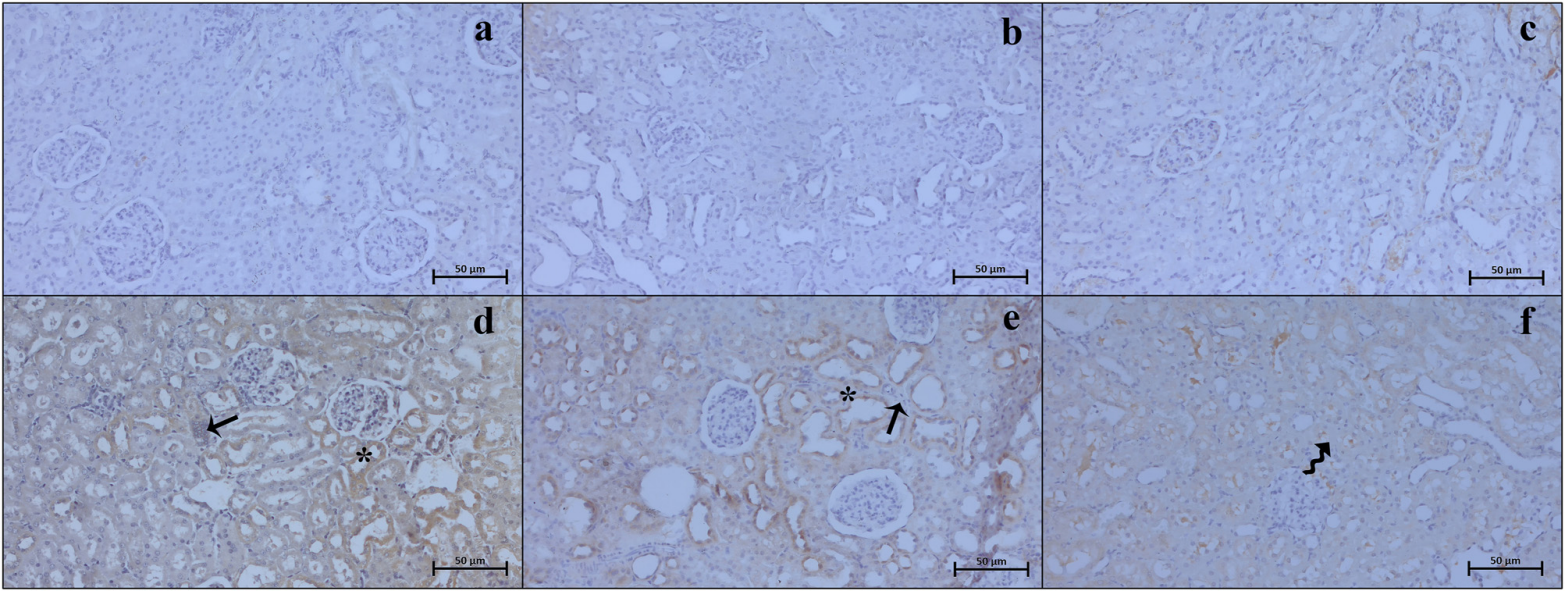
Representative TNF-α immunohistochemistry micrographs of control (a), Visnagin30 (b), Visnagin60 (c), I/R (d), I/R + Visnagin30 (e), and I/R + Visnagin60 (f) groups. In the I/R exposed animals, tubular (arrow) and glomerular (asterisk) Caspase 3 expression is significantly increased. The immunodensity is significantly alleviated in parenchyma of visnagin treated animals (curved arrow). Staining: TNF-α IHC. Counterstain: Hematoxylin. Bar: 50 µm.

**Figure 6:**
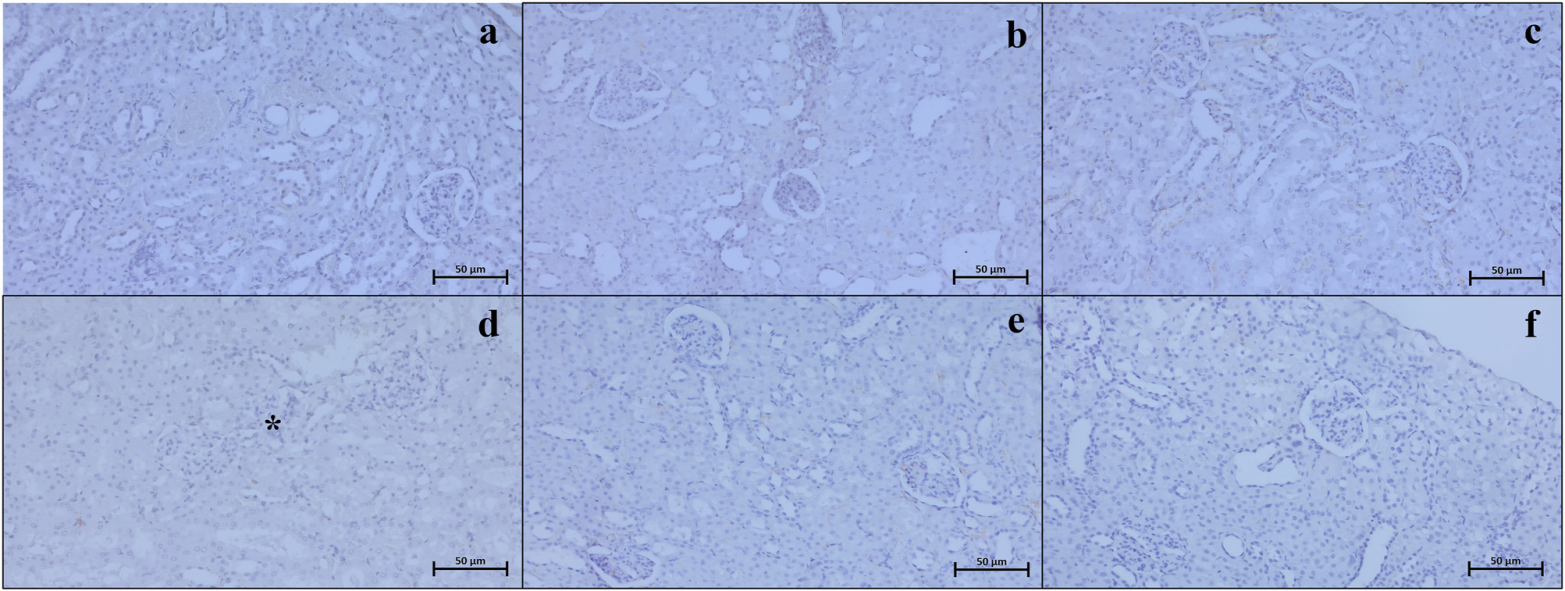
Representative CD86 immunohistochemistry micrographs of control (a), Visnagin30 (b), Visnagin60 (c), I/R (d), I/R + Visnagin30 (e), and I/R + Visnagin60 (f) groups. The CD86 immunodensity is slightly upregulated in parenchyma of I/R group (asterisk). Staining: CD86 IHC. Counterstain: Hematoxylin. Bar: 50 µm.

**Figure 7:**
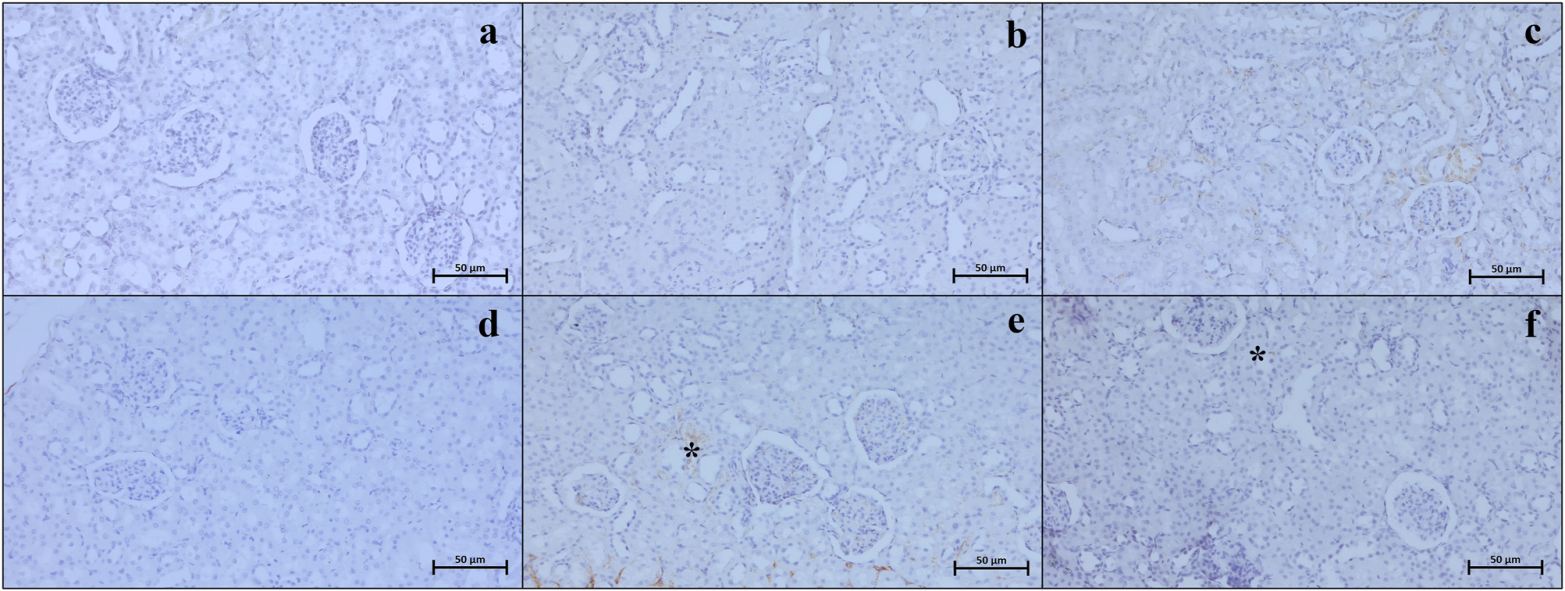
Representative CD163 immunohistochemistry micrographs of control (a), Visnagin30 (b), Visnagin60 (c), I/R (d), I/R + Visnagin30 (e), and I/R + Visnagin60 (f) groups. Immunodensity of CD163 is slightly but significantly upregulated in tubular and glomerular structures (asterisk) in visnagin treated animal kidney. Staining: CD163 IHC. Counterstain: Hematoxylin. Bar: 50 µm.

Our examinations revealed that the Bax (Figure 3), Caspase 3 (Figure 4), TNF-α (Figure 5), CD86 (Figure 6), and CD163 (Figure 7) proteins, which were analyzed through immunohistochemistry, were expressed at low levels in the renal tissue. These proteins were observed not only in the cells located within the renal stroma but also sporadically in different cells of the parenchymal kidney tissue and even in the extracellular matrix.

Statistical analysis indicated that the expression levels of Bax, Caspase 3, TNF-α, CD86, and CD163 proteins were similar among the Control, Visnagin30, and Visnagin60 groups (p>0.05). However, in the I/R group, there were significant increase in the levels of Bax, Caspase 3, TNF-α, and CD86 with respect to the control group (p<0.001, p<0.05, p<0.05, p<0.0.001, respectively). The CD163 immunodensity in I/R group was not significant changed according to the control group (p>0.05). Visnagin treatment with 30 mg/kg did not change the immuodensity of Bax, Caspase 3, TNF-α, CD86, and CD163 proteins in I/R + Visnagin30 group with respect to the I/R group significantly. But administration of Visnagin with a single dose of 60 mg/kg reduced the levels of Bax (p<0.001), Caspase 3 (p<0.01), TNF-α (p<0.01), and CD86 (p<0.05) proteins and increased the CD163 (p<0.05) protein immunodensity in I/R + Visnagin60 group compared to the I/R group, significantly (Table 2).

**Table 2:** Statistical analysis of the Bax, Caspase3, TNF-α, CD86 and CD163 immunodensity in renal tissues of the groups.

	Control	Visnagin30	Visnagin60	I/R	I/R + Visnagin30	I/R + Visnagin60
Bax immunodensity, %	11.18 ± 2.38	13.04 ± 3.53	11.91 ± 2.50	22.74 ± 10.21^b^	19.47 ± 10.48	12.08 ± 2.86^+++^
Caspase 3 immunodensity, %	6.27 ± 1.18	5.49 ± 1.33	5.91 ± 1.63	7.75 ± 2.55^a^	6.51 ± 1.83	6.11 ± 1.56^++^
TNF-α immunodensity, %	14.28 ± 4.29	13.44 ± 3.83	14.33 ± 5.04	18.90 ± 7.95^a^	17.86 ± 6.63	13.62 ± 4.39^++^
CD86 immunodensity, %	2.99 ± 1.02	3.16 ± 0.98	2.98 ± 0.98	6.45 ± 3.39^b^	7.40 ± 3.57	4.34 ± 2.57^+^
CD163 immunodensity, %	3.38 ± 0.94	2.89 ± 1.10	3.41 ± 1.13	2.48 ± 1.35	3.32 ± 1.07	3.54 ± 1.47^+^

Data are shown as Mean±SD ^a^p<0.05, ^b^p<0.001 vs. respective control group. ^+^p<0.05, ^++^p<0.01, ^+++^p<0.001 vs. respective I/R group.

## Discussion

This study demonstrated that visnagin administration significantly attenuated renal ischemic-reperfusion injury, reduced oxidative stress and inflammation, increased M2 macrophage polarization, and suppressed apoptosis. Specifically, visnagin administration at a dose of 60 mg/kg significantly reduced I/R-induced histopathological and biochemical damage.

In our study, serum BUN and creatinine levels were observed to increase significantly in the I/R group and the visnagin-administered I/R+visnagin groups following bilateral renal ischemia and 2 h of reperfusion. These findings indicate that renal I/R injury leads to acute kidney dysfunction, reflected by increases in biomarker levels. Our results are consistent with the findings of Younis et al., reported significant increases in BUN and creatinine levels in the I/R and vehicle groups using a 30-min bilateral renal ischemia and 2-h reperfusion rat model [28]. Furthermore, a study by Hameed Ali et al. also found significant increases in renal function parameters after 2 h of reperfusion, and this was associated with a decrease in glomerular filtration rate [29]. These data suggest that a 2-h reperfusion period is sufficient to detect early renal dysfunction. Consistent with the literature, our study also observed significant deterioration in renal function indices in the I/R-exposed groups, which was considered a biochemical indicator of acute kidney injury. However, longer reperfusion times are needed for a comprehensive assessment of chronic kidney injury and regenerative processes. This study supports the suitability of a 2-h reperfusion period for analyzing early biochemical and histopathological changes.

Reduced blood flow to the kidney due to ischemia is a clinical complication. Renal ischemia can be observed in the patients suffering from the diseases such as renal artery stenosis, hypotension, and sepsis. Furthermore, renal ischemia can be encountered iatrogenically during kidney transplantation or partial nephrectomy [30]. Previously published articles highlighted the impact of reactive oxygen species (ROS) and nitric oxides in renal injury during renal I/R process [31], 32]. These studies also concluded that the potential of targeting ROS to alleviate severity of the I/R injury, and even organ recovery. Current literature indicates the antioxidants, mainly plant sourced, are potential promising co-modalities in recovery of the injured kidney with modulating various cellular signaling [33]. Although there is no data for the reno-protective potential of visnagin in renal I/R injury, this furanochrome’s promising results are reported in I/R injury in various organs such as hearth, brain, and testis [15], 18], 34]. Our observations are consistent with the current literature which are reporting antioxidant, anti-inflammatory and anti-apoptotic properties of this drug in I/R injury. For example, in a previously published article, Rao et al. indicated that the upmodulation of MDA, inflammatory IL-1β, IL-6, and TNF-α, apoptotic Bax and Caspase 3, but downregulated GSH, superoxide dismutase (SOD) and catalase (CAT) activity are improved through visnagin treatment in cerebral I/R injury [34]. Although tissue of our current study and Rao et al. are varying, the main results and promising observations of visnagin are consistent. The number of the studies reporting potent miraculous properties of visnagin in I/R injury can be increased. Fu et al. and Sagir et al. reported organoprotective function of visnagin in cardiac and testicular I/R injury with modulating cellular oxidative stress, apoptosis and inflammation consistently as our observations [15], 18]. When considered as a whole, the current literature and our current study are highlighting visnagin treatment during ischemic conditions may regulate imbalaced homeostatic cellular conditions such as inflammatory (TNF-α and IL-1β), apoptotic (Bax and Caspase 3) oxidative stress (MDA, luminol and lucigenin CL Assay) markers, which increased after I/R injury.

Although these observations indicate the potential renoprotective profile of visnagin, the underlying cellular regulation has not been fully understood yet because of complexity of this process. The plenty of reported studies indicate and reminds the complex process of this clinical condition. Although our study demonstrated that Vis modulates inflammation, apoptosis, and oxidative stress, the molecular signaling pathways underlying these effects (specifically PI3K/Akt, JAK/STAT, and NF-κB) could not be directly investigated experimentally within the scope of our study. Existing literature suggests that these pathways play important roles in renal ischemic-reperfusion injury [35], 36]. Xie et al. demonstrated that specific cellular signalings such as PI3K/Akt are taking role during cellular injury and recovery of the kidney in I/R injury [35]. In another study, Zhao et al. reported the role and JAK/STAT sgnaling during renal I/R injury [36].

Previous studies have shown that visnagin modulates inflammation and apoptosis through various cellular signaling pathways. In particular, suppression of NF-κB signaling has been linked to visnagin’s anti-inflammatory effects in both microglial cells and cardiac and pancreatic tissues [17], 37], 38]. Furthermore, visnagin has been reported to exert regulatory effects on the PI3K/Akt/mTOR axis, thereby controlling cell proliferation and apoptosis [39]. The PI3K/Akt pathway plays a decisive role in cellular survival, anti-apoptosis, and redox balance. It regulates NF-κB and Bcl-2 through the Akt protein. Activation of this pathway may limit cell death by inducing antioxidant systems and shifting the Bax/Bcl-2 balance in an anti-apoptotic signaling pathway [40]. Visnagin’s decreased Bax expression and its effects on other apoptotic proteins expression in our study may be related to this pathway. Similarly, JAK/STAT3 plays a role in the expression of proinflammatory cytokines. Activation of STAT3 by cytokines such as IL-6 via JAK2 may increase inflammatory responses in renal tissue, leading to tissue damage [41]. Visnagin’s decreased levels of both IL-6 and TNF-α in our study may indicate suppression of JAK/STAT3 signaling. Furthermore, the NF-κB pathway is a major regulatory mechanism of I/R injury. The NF-κB signaling pathway plays a central role in the initiation of the inflammatory process. Oxidative stress and proinflammatory stimuli activate NF-κB, increasing the transcription of mediators such as TNF-α, IL-1β, and COX-2 [42]. A study by Tian et al. showed that visnagin administration reduced NF-κB activity and COX-2 expression via prostaglandin E2, thereby providing a protective effect against ischemic brain injury in rats [43]. Decreased IL-1β levels and decreased TNF-α expression in our study through visnagin may also mediate the inhibition of NF-κB activity. The decrease in proinflammatory cytokines and apoptotic proteins observed in our study suggests that visnagin reduces renal tissue damage through these signaling pathways. However, more comprehensive studies are needed to better understand the precise cellular mechanisms of visnagin in renal I/R injury.

Even though the role of inflammation during I/R injury and anti-inflammatory properties of visnagin are discussed above, the background of this process is more complicated than predicted. Most of the studies are reporting up-modulated pro-inflammatory cytokine levels in injured kidney, but the data based on the macrophage polarization is very limited. Previously published papers reporting crucial role of M1/M2 macrophage polarization during the injury period and post-damaged recovery process [44]. In an experimental study, Jiang et al. indicated the role of M1/M2 macrophage polarization during the modulation of inflammatory process in renal I/R injury [45]. Furthermore, the authors reported a plant sourced flavone, luteolin, modulate M1/M2 macrophage polarization to the M2 macrophage side. This study indicated that renal ischemia resulted with the increased expression of M1 macrophage phenotype markers such as IL-6, TNF-α, CD16/32. Results of this study also demonstrated that the antioxidant drug administration suppressed M1 macrophage phenotype markers, but upmodulated M2 cellular indicators such as IL-10 and CD206. Although examined polarization markers of this study and our current study are varying, the reached results are consistent. Besides of the reported macrophage polarization markers by Jiang et al., we reported that renal I/R injury significantly increased M1 macrophage phenotype markers of IL-1β, IL-6, and TNF-α. Results of this study also revealed that M1 cell surface marker of CD86 expression is dramatically increased. In addition, antioxidant treatment of visnagin modulated the macrophage polarization to the M2 side mainly in the higher dose exposed animals. Resuts of this study reporting the reduce of M1 macrophage markers, but dramatically increased M2 macrophage specific cell surface markers of CD163 in visnagin treated group. Elevated levels of inflammatory factors hinder the healing process in I/R-induced renal injury and are associated with increased expression of M1 markers and decreased expression of M2 markers [46].

Previous studies have reported that some natural antioxidant compounds exhibit similar protective effects in I/R injury by suppressing apoptosis and promoting healing. For example, naringin and trimetazidine have been shown to provide renoprotective effects against renal I/R injury by downregulating microRNA-10a and inhibiting apoptosis [47]. Furthermore, a recent review of plant antioxidants reports that these substances act on apoptosis signaling and inflammatory processes and have therapeutic potential in I/R-associated tissue damage [48]. Based on these studies, visnagin’s effects on apoptosis and inflammation are similar to those of other antioxidant agents and could be considered a novel therapeutic target.

Evidence suggests that preventing the initial influx of macrophages can mitigate I/R mediated renal damage [49]. Early depletion of macrophages leads to reduced renal injury, while their removal at later stages impairs recovery, indicating that macrophage polarization plays a critical role in the recovery process following I/R induced renal injury [50]. It was also reported that the injured tubular epithelial cells (TECs) can activate M1 macrophages in renal injury [51]. Thus, the polarization of macrophages and the maintenance of cellular homeostasis may contribute to the recovery from renal injury following I/R. In a study by Tian et al., the anti-ischemic effect of visnagin was evaluated in male rats. Middle cerebral arterial ischemia/reperfusion was induced and treated with different concentrations of visnagin. The neuroprotective effect of visnagin against cerebral ischemia was assessed by analyzing various pathological observations. The anti-inflammatory property of visnagin was evaluated by measuring the levels of pro-inflammatory cytokines in the serum and brain tissues of cerebral ischemic rats. Prostaglandin E-2, COX-2, and NFκ-β were also measured to assess the anti-ischemic effect of visnagin [43]. A substantial body of evidence suggests that the conversion of resident tissue macrophages from a pro-inflammatory M1 phenotype to an anti-inflammatory M2 phenotype following acute trauma and I/R injury accelerates healing and promotes tissue neoangiogenesis [52]. To our knowledge, our study is the first to investigate the effect of visnagin on M1/M2 polarization in renal ischemia-reperfusion injury, and we believe it contributes to the literature in this regard. Our findings indicate that, particularly in the high-dose exposed group, oxidative stress markers were similar as measured in control group. These findings suggest that visnagin may have a potent to protect renal tissue and parenchyma in I/R injury by contributing various cellular signalings and regulating expression of various apoptotic, inflammatory proteins. Moreover, visnagin posing a promising potential to regulate the destiny of the macrophages.

## Limitations of this study

Although this is the first study reporting beneficial property of visnagin on renal I/R injury, there are some issues that should be considered. We examined potential nephroprotective effect of this drug in renal I/R injury in a short period and the lack of the results based on the chronic protective properties of this drug reveals new questions to be addressed. Besides of the methodological weak sides, we examined tissue level of some biochemical and histopathological results, but the absence of laboratory analyses such as RT-qPCR and immunoblotting detections methods like western blotting would also be used to confirm the results and understand the correlations between gene and proteins to explore the underlying mechanisms more clearly. However, as a novel study, our observations indicate the potential protective activity of this drug with modulating oxidative stress, apoptosis, inflammation and possible role of M1/M2 macrophage polarization in this process, but we still need more studies to understand the complex process of renal I/R injury and the appropriate response for how the visnaging poses its protective activity in this process.

## Conclusions

In conclusion, visnagin is a potential compound that demonstrates protective effects in renal ischemia-reperfusion injury and can protect renal tissue by modulating M1/M2 macrophage polarization. This study highlights the potential of visnagin’s anti-inflammatory and antioxidant properties in preserving renal function. Although our observations in the I/R + Visnagin30 and I/R + Visnagin60 groups suggest that this organic chemical does not exhibit nephrotoxic effects at the doses administered, future comprehensive studies should focus on establishing detailed protocols necessary for the clinical use of visnagin and conducting more in-depth analyses of its protective molecular dynamics in ischemia-reperfusion injury and more studies are required to explore clinical applicability of Visnagin in testicular I/R injury.
